# Breeding for beneficial microbial associations

**DOI:** 10.1038/s41467-026-76260-6

**Published:** 2026-08-03

**Authors:** Ido Rog, Stefanie Lutz, Lukas Fesenfeld, Jürg Hiltbrunner, Harro Bouwmeester, Marcel G. A. van der Heijden

**Affiliations:** 1 Department of Evolutionary Biology and Environmental Studies, University of Zurich, Zurich, Switzerland; 2 Agroscope, Zurich, Switzerland; 3 Einstein School of Public Policy, Political Economy of Technology Group, ETH Zurich, Zurich, Switzerland; 4 Competence Division Plant and Plant Products, Research Group Extension Arable Crops, Agroscope, Zurich, Switzerland; 5 Plant Hormone Biology Group, Swammerdam Institute for Life Sciences, University of Amsterdam, Amsterdam, Netherlands

**Keywords:** Environmental sciences, Sustainability, Plant breeding, Soil microbiology, Agriculture

## Abstract

Beneficial plant–microbe associations (BMAs) offer a valuable opportunity to reduce dependence on synthetic inputs. However, traditional breeding has rarely targeted traits that enhance beneficial interactions, and conventional agricultural practices have often degraded soil health and microbial diversity. We present a framework that combines breeding for traits that facilitate BMA with soil management practices that enrich BMA. This approach integrates advanced breeding technologies with strategies for precise production and inoculation of microbes. Strengthening these complementary plant- and microbe-centered approaches and encouraging their adoption by farmers can foster more resilient and productive agricultural systems with reduced dependence on pesticides and fertilizers.

## Abstract (web summary)

Traditional crop breeding has rarely targeted traits that enhance beneficial plant–microbe associations (BMAs). Here the authors propose a framework that combines breeding for traits that facilitate BMA with soil management practices that enrich BMA.

## Introduction

Agriculture today faces significant challenges in meeting the growing demand for food in a rapidly changing world. Since the onset of the agricultural revolution, farming systems have increasingly relied on high-yielding crop varieties, genetically resistant cultivars, synthetic fertilizers, and pesticides to maximize productivity. While these inputs have substantially increased food production, the intensification of agricultural practices has often come at the expense of long-term soil health^1^ and accumulation of pesticides^2^.

Soil is the most biodiverse habitat on land^3^ and contains a plethora of micro-organisms, many of which can enter beneficial but also pathogenic associations with plants. Optimizing plant-beneficial microbial associations (BMA) can significantly enhance plant performance by improving nutrient acquisition, promoting growth, and increasing resistance to both abiotic and biotic stresses^4–6^. While numerous papers have reviewed the mechanisms by which BMAs support plant health and growth^7–10^ there remains a critical gap in translating this knowledge into practical strategies for real-world agriculture to promote sustainable agriculture.

Microorganisms are key drivers of ecological processes across a wide range of environments and play essential roles in human health and food production^11^. One of the most extensively studied groups of beneficial microbes are the arbuscular mycorrhizal fungi (AMF), which form mutualistic associations with approximately two-thirds of all terrestrial plant species, including many crops. Through their extensive hyphal network, AMF significantly expand the effective root zone, enhancing water and nutrient uptake—up to 40–90% for nitrogen and phosphorus, respectively—and thereby improving plant growth and resilience^12^. Nitrogen-fixing bacteria (e.g., *Rhizobia*) represent another vital group of symbiotic microbes in agroecosystems. These bacteria engage in efficient mutualisms with mostly leguminous host plants, wherein the plant supplies photosynthetically derived organic carbon in exchange for biologically fixed nitrogen. This interaction is highly beneficial, as over 90% of the nitrogen fixed by these bacteria is directly utilized by the host plant^13,14^.

Not all beneficial microbes form direct symbiotic relationships with plants. A substantial group of non-symbiotic but plant-associated beneficial organisms includes Plant Growth-Promoting Bacteria (PGPB), Plant Growth-Promoting Fungi (PGPF), and Plant Growth-Promoting Protists (PGPP)^15–17^. These groups are not defined by specific taxonomic classifications or universal functional traits, but rather by their capacity to enhance plant growth under certain environmental conditions. While native communities of beneficial microbial symbionts are usually present, their abundance and functional activity are often diminished in intensively managed agricultural soils^18,19^. Therefore, strategies to restore and promote BMAs to unlock their full potential for sustainable crop production are urgently needed.

**Fig. 1:**
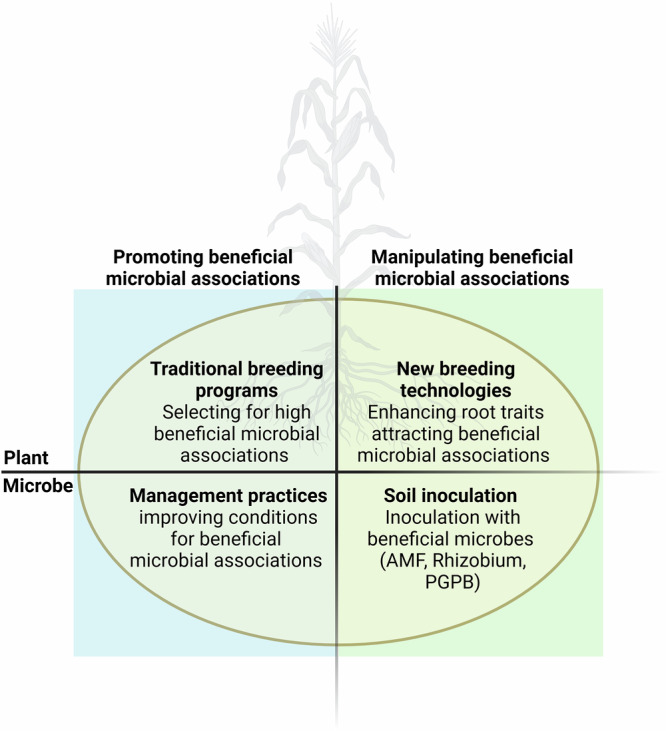
A dual ecological engineering approach to enhance sustainable agriculture. Plant- and microbe-centered strategies represent a dual ecological engineering framework to improve plant health, soil fertility, and long-term agricultural sustainability. The figure is organized along two axes: the horizontal axis separates plant-based interventions (top row) from the microbial-based approaches (bottom row), while the vertical axis distinguishes strategies aimed at promoting beneficial associations (left) from those that manipulate associations (right). **Top left**: Traditional breeding programs aimed at crop genotypes with enhanced beneficial associations. **Bottom left**: Management practices that improve soil conditions and promote native beneficial associations. **Top right**: New breeding technologies that enhance root traits and manipulate exudation of small molecules to improve recruitment of beneficial microbes. **Bottom right**: Soil microbiome manipulation through inoculation with beneficial microbes such as arbuscular mycorrhizal fungi, Rhizobium or plant growth-promoting bacteria (PGPB). Created in BioRender. Golan davydov, O. (2026) https://BioRender.com/ t0cub0y.

Beneficial plant-microbe interactions could be enhanced from both sides of the mutualistic relationship: either through plant-centered approaches or microbial community manipulation. While a range of recent reviews discussed how plant or microbe engineering can help to boost agricultural sustainability, little attention has been given to this combined approach (Fig. 1). Combining both plant genetic selection and microbial community management has the potential to create synergistic effects, leading to more stable and effective BMAs.

Here, we aim to highlight the potential of plant BMAs and the strategies available to enhance these interactions to improve ecosystem performance. On the plant side, we review recent advances in breeding programs that focus on selecting genotypes with enhanced capacity for microbial recruitment, particularly those aiming to alter root exudation such that the plant becomes more conducive to beneficial microbial colonization. On the microbial side, we discuss emerging soil management practices aimed at supporting native beneficial microbes, as well as innovative field inoculation techniques using microbial consortia. In addition, we review the signaling molecules and root traits involved in initiating and sustaining BMA. Finally, we address a crucial component of implementation: the acceptance of these approaches by farmers and citizens. We discuss the sociocultural and practical barriers to adoption and propose future directions to facilitate the translation of this technological framework into a farmer-adopted, scalable, field-ready strategy for sustainable agriculture.

## Traditional breeding programs can enhance microbial association

Effective BMA enhancement requires a supporting root system. A central open question is which specific root traits influence the assembly of the rhizosphere microbial community and promote beneficial associations^20^. A promising plant breeding approach not only focuses on resistance genes and susceptibility genes against pathogens, but also includes microbiome genes (*M* genes)^21^ that facilitate and regulate plant-microbiome interactions. Recently, a new framework for implementing *M* genes in plants was proposed, with the potential to increase agricultural sustainability^21^.

To date, relatively few breeding efforts have focused on enhancing plant traits that promote beneficial interactions with microbes. However, the concept of integrating microbial benefits into agroecosystems, referred to as *ecological engineering*, has gained considerable attention in recent years, particularly as a strategy to support sustainable agriculture^22^. Previous studies have demonstrated a link between plant genetic background and traits associated with beneficial microbial interactions^23^. Modern varieties, however, may have lost the capacity to form mutualistic associations with microbes, and consequently, can benefit less from BMA^24^. Classical breeding approaches offer the potential to restore these interactions by selecting traits from wild genotypes that still possess the gene loci necessary for plant–microbe associations. Strengthening such interactions could help in enhancing BMA associations.

Modern crop breeding has primarily focused on achieving high yields, resistance to biotic and abiotic stresses, and efficient nutrient uptake. However, these traits are typically evaluated under conditions of sufficient nutrient availability, pest and disease control. Also, breeders have focused on avoiding the negative effects of pathogens, rather than strengthening the positive interactions of crops with microbes. As a result, over years of domestication and selective breeding, physiological characteristics such as larger seed size, improved palatability, and increased biomass allocation to the agricultural product have been favored. Due to the high nutrients availability in the breeding trails plants allocate less resources to the roots, possibly at the expense of traits that support beneficial microbial associations involved in nutrient and water acquisition^25,26^. Several genetic mechanisms may explain BMA disruption, including direct or indirect artificial selection, the accumulation of deleterious mutations associated with breeding population demographics, or the selective neutrality of these traits under high-nutrient conditions^27^. In view of the predicted changes in climate extremes, including drought, future breeding programs should specifically focus on the root system architecture and select for specific below-ground traits directly. This is especially important as the root system architecture plays a major role in the BMA, where specific traits are hypothesized to enhance the beneficial microbes^28^. To optimize BMA, breeding programs need to select for specific below-ground traits directly.

Breeding programs targeting BMA may involve important trade-offs. Modern breeding programs primarily focus on maximizing yield and yield stability, whereas enhancing BMA may require increased carbon allocation to support microbial symbionts^29,30^. Although selection for enhanced BMA could improve performance under low-nutrient conditions, it may reduce productivity in high-input agricultural systems^31,32^. In addition, selecting for belowground traits could be genetically or physiologically linked to undesirable agronomic characteristics, including variation in floral traits and pollinator interactions^33^. When breeding for specific beneficial microbes, it is important to verify that such breeding targets do not also facilitate specific plant pathogens (e.g., that may benefit from specific root exudates or changes in the overall microbiome). Nevertheless, despite the strong potential of BMA for sustainable agriculture, breeding efforts should continue to prioritize overall plant performance and agronomic fitness.

## New breeding technologies enhance beneficial associations

In addition to leveraging natural selection for BMA from wild relatives, new breeding techniques (NBT) offer new opportunities to enhance BMA. The overall approach of harnessing the plant genome to shape the microbiota was demonstrated in the rice phyllosphere by targeting specific plant metabolites, resulting in increased protection against pathogens^34^. Subsequently, specific *M* genes were used to improve plant growth and productivity through the modulation of beneficial plant–microbe interactions^35^. For example, modifications in lignin precursor metabolism were shown to alter the endosphere bacterial microbiome^36^ and enrich beneficial microorganisms in the rhizosphere^37^. While several studies have focused on individual *M* genes^38,39^, many of these traits are regulated by complex gene networks, and the secretion of signaling compounds often involves additional genes, making breeding strategies considerably more complex.

**Fig. 2:**
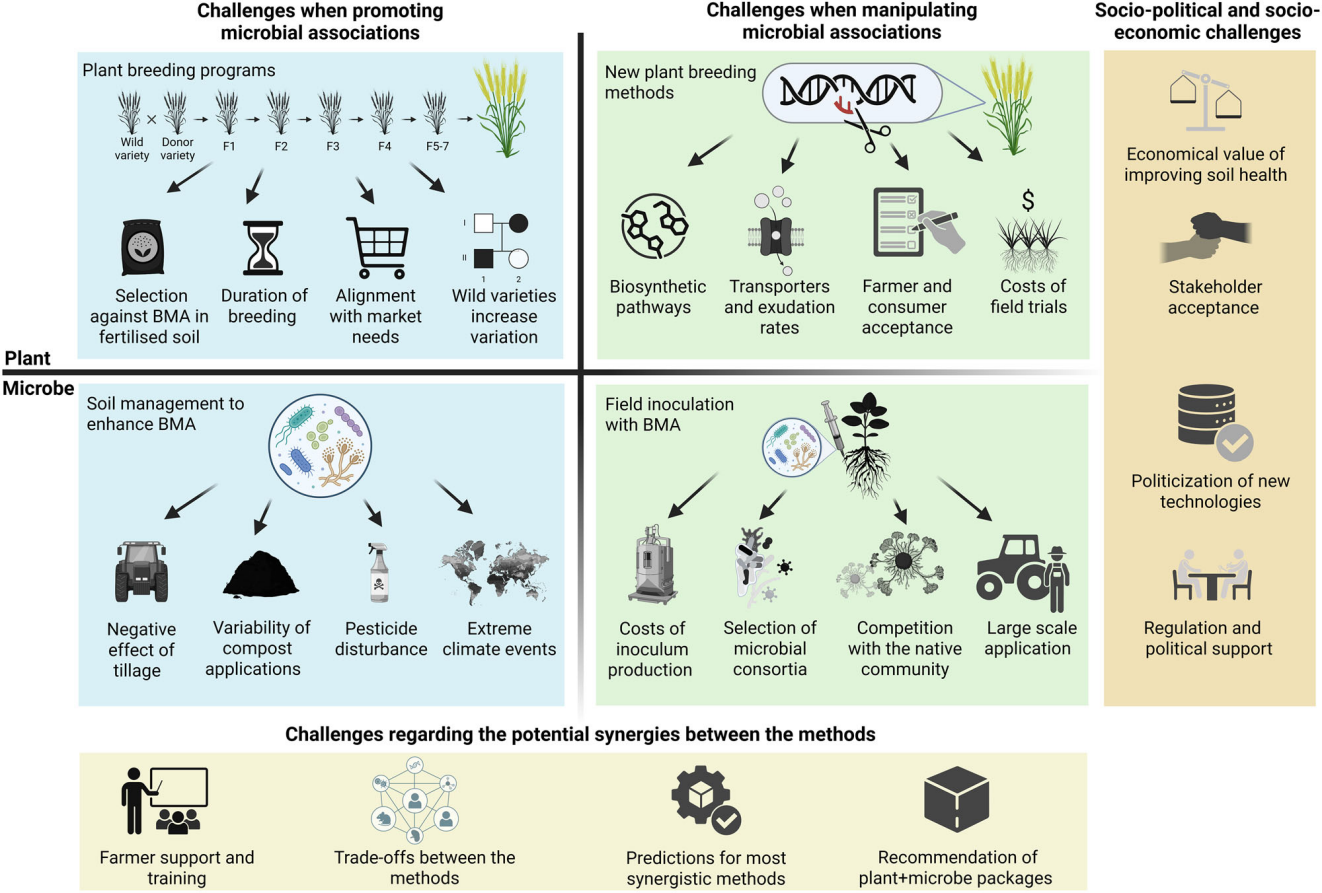
Challenges of the dual ecological engineering approach. The left side outlines key challenges related to promoting beneficial microbial associations, while the right side presents challenges associated with manipulating beneficial microbial association strategies. The top row highlights interventions and limitations from the plant side (e.g., breeding and genetic engineering), and the bottom row focuses on microbial-side challenges, including inoculation practices, microbiome compatibility, and soil variability. Addressing these challenges is essential for the successful integration of beneficial microbial associations (BMA) into diverse agricultural systems. On the right side, we addressed the socio-economic and socio-political challenges to scale new technologies. On the bottom the challenges in addressing the potential synergies between the methods. Created in BioRender. Golan davydov, O. (2026) https:// BioRender.com/9nsp62e.

One approach is to directly manipulate the small molecules involved in recruiting beneficial microbes. Plants release approximately 20–40% of their assimilated carbon into the rhizosphere as a complex blend of signaling compounds, including amino acids, fatty acids, and other metabolites^40^. These exudates not only attract beneficial microbial groups but also help repel pathogenic organisms^41,42^ (Fig. 2). Mutation within some metabolic pathways has been shown to alter the composition of the root exudate (Supplementary Table 1), thereby also reshape the microbial community^43,44^. Parallel to the innovation in pathways elucidation, technological advancements have improved the ability to detect and analyze root exudates, even under field conditions^45^, although the detection of microbiota recruitment signals remains challenging as they are produced in low concentrations^46^. Through the application of NBT, it is now becoming feasible to modify specific metabolic pathways and possibly enhance the recruitment of targeted beneficial microbial groups.

The second approach, complementary to root exudation, is root system architecture and molecular structure, which are crucial to BMA. Architectural traits, such as root depth and root density, along with morphological features like root diameter and root hairs, determine the root’s capacity to engage with beneficial microbes^47,48^. At the molecular level, nutrient transporter expression is essential for symbiotic interactions. For instance, phosphate transporters are strongly expressed during AMF associations^49^, while ammonium and nitrate transporters play crucial roles in rhizobia symbiosis^50^. In addition, the transfer of organic carbon to symbionts, either in the form of sugars (e.g., via sugar transporters^51^) or fatty acids^52^, is a key molecular trait supporting mutualism. Moreover, it is important to mention that root exudates comprise thousands of different substances, and the inclusion or exclusion of specific compounds provides opportunities to support or suppress specific groups of microbes. This plant-microbe morphological crosstalk offers an opportunity to enhance BMAs through targeted intervention.

To date, only two genetically engineered traits, herbicide tolerance and insect resistance, have been widely adopted in crops, both demonstrating substantial environmental and economic impacts globally^53^. In contrast, NBT plants targeting BMA traits have received limited attention, despite their significant potential to improve soil health and promote sustainable agriculture. Enhancing naturally occurring traits within the plant genome—rather than introducing entirely new gene loci—may also increase the likelihood of acceptance among farmers, policymakers and the public. The application of CRISPR-Cas9 and related gene-editing technologies enables precise manipulation of signaling pathways involved in microbe recruitment. By enhancing the production of specific signaling molecules or modifying the composition of root exudates, it is now feasible to develop NBT genotypes that optimize BMA^46^. Several signaling compounds, including strigolactones, flavonoids, and benzoxazinoids, have already been identified as key mediators in the recruitment of beneficial microbes. However, most of this research remains confined to laboratory or greenhouse conditions, and comprehensive field trials and long-term studies are still lacking. Future research should focus on testing and refining NBT genotypes under field conditions to ensure agronomic viability and cost-effectiveness for real-world agricultural systems.

## Management practices improving beneficial microbial associations

Scientists and farmers have developed a variety of management practices to enhance crop yields and soil health, many of which directly or indirectly improve conditions for BMA. In recent years, two overarching systems of agricultural management have emerged—organic and conventional practices. Organic farming has become one of the most widely adopted approaches to improving soil multifunctionality, primarily through the use of natural pesticides and fertilizers instead of synthetic inputs^54,55^. In addition, organic practices often incorporate cover cropping and more diverse crop rotations, which can significantly influence soil functions and microbial dynamics^56^ (Supplementary Table 2). While current agricultural management tends to focus heavily on abiotic factors such as soil organic carbon and nutrient availability, greater emphasis should be placed on practices that foster the abundance and activity of beneficial microbial associations. Promoting BMA can play a crucial role in enhancing long-term soil health, plant resilience, and sustainable productivity.

Microbial diversity—and in particular, BMA—has been shown to enhance primary productivity^19^. However, it remains unclear whether increasing microbial diversity through specific agricultural practices can directly improve soil functioning and promote crop yield or indirectly via changes in soil properties. One promising strategy to enhance microbial diversity involves modifying the plant community structure and implementing crop rotation. Increasing both the functional and taxonomic diversity of plant communities has been shown to boost microbial diversity^57^ and improve nutrient availability for plants^58^. Diverse plant communities influence the rhizosphere through multiple pathways: the quality and quantity of root and leaf litter, and the exudation of specific compounds and signaling molecules. Moreover, certain plant groups are known to engage more readily in BMA, such as AMF and nitrogen-fixing bacteria, thereby enriching the soil microbiome. For instance, *Brassica* species are typically non-mycorrhizal hosts, but are used for soil biofumigation^59^. In contrast, legumes are well-known for forming symbiotic relationships with both AMF and nitrogen-fixing bacteria and enhance the abundance of BMA. Crop rotation can also play a role in disease suppression by disrupting the life cycles of plant-specific pathogens through the strategic inclusion of non-host species^60^. Importantly, the composition of the soil microbial community may ultimately have a greater influence on disease resistance and soil function than microbial diversity alone^61^. Understanding these complex plant–microbe–soil interactions is essential for designing cropping systems that promote sustainable productivity.

Tillage practices are widely employed by farmers for weed control, soil aeration, and to prepare favorable seedbeds. However, intensive tillage poses several risks to BMA. Conventional tillage can damage soil structure, accelerate nutrient loss, increase carbon emissions, soil erosion, reduce microbial diversity, and disrupt soil microbial networks. As a response, *reduced-tillage* and *no-tillage* systems have gained popularity and are now practiced on approximately 12.5% of the world’s arable land^62^. These conservation tillage approaches significantly alter the biomass and composition of soil microbial communities^63^. Moreover, integrating organic management with reduced tillage has been shown to improve soil structure, reduce erosion, and enhance the abundance and functional complexity of beneficial microbes^64–66^. A holistic approach that includes cover crops—especially mycotrophic species that host AMF—can further enrich the soil with BMA and amplify the advantages to crops. Since agricultural management encompasses multiple interacting practices, integrating strategies that protect and promote BMA is essential for advancing sustainable farming systems.

## New technologies of effective microbe manipulation

Field inoculation with BMA offers a promising strategy to restore microbial abundance, reshape the soil microbial community, and enhance plant performance. Despite its high potential, developing effective microbial inoculants for large-scale field application presents several significant challenges (Fig. 2). One major obstacle is producing inoculants that consistently deliver beneficial outcomes across varying climates, soil types, and crop systems^67^. In general, native inoculants are more promising than foreign inoculants and have a more substantial effect on many parameters^68^. Inoculants must be capable of surviving and competing with native soil microbial communities, which often limit their establishment and long-term effectiveness. Another critical challenge lies in producing high concentrations of viable microbial propagules in a cost-effective and reproducible manner^69^. These challenges, combined with the high potential of BMA inoculation, have created a growing commercial market^70^. However, despite the size of the market and the promise of microbial technologies, product quality has often fallen short of expectations, with many formulations lacking consistent viability and effectiveness^70^. This gap highlights the urgent need for the development of more robust, reliable, and biologically active microbial inoculants to support sustainable agriculture. Also, procedures for product quality control and market entry regulation need further attention.

In recent years, microbial inoculation products have shifted from single-strain formulations to microbial consortia^71^. The goal of consortia inoculation is to enhance effectiveness across a broader range of environmental conditions compared to single-strain inoculants. Traditionally, the development of single-strain inoculants involved a linear process: isolation, identification, characterization, and field application^71^. In contrast, the development of microbial consortia requires an additional layer of complexity, including the selection of microbes based on individual and community-level functional traits, as well as their performance within microbial networks^72^. Recent perspectives emphasize that successful consortia should mimic natural microbial communities, incorporating complementary interactions among microbial members^72–74^. Advanced synthetic microbiomes have already been developed using modeling-based frameworks to design specific functions, by assembling microbial consortia with targeted traits^75^. Furthermore, this evolving approach calls for a plant-centric perspective, considering plant genetic diversity and its influence on plant–microbe and plant–plant interactions^76,77^. These interconnected dynamics are essential for the rational design of next-generation microbial inoculants that are effective, resilient, and compatible with a wide range of crop genotypes and soil environments.

Several technologies have been developed to ensure the viability and efficiency of microbial inoculants at low production costs. Despite the well-documented benefits of BMA, the adoption of these technologies has remained relatively slow. One effective approach is seed-applied microbial inoculation, such as microbial coatings, which deliver microbes directly to the seed and enhancing root colonization. In parallel, there has been a growing interest in alternative delivery systems such as granular, liquid and biochars inoculants^78^. These methods are particularly well-suited to developed agricultural systems, where sowing machinery can be modified to dispense bio-inoculants alongside fertilizers and pesticides. While notable advancements have been made in the formulation and delivery technologies for microbial inoculants, much of the innovation is protected by patents and remains inaccessible to the broader scientific community. Greater transparency and standardization would accelerate research, foster innovation, and improve the overall effectiveness and adoption of BMA-based solutions.

## Enhancing beneficial associations under varied soil health status

The link between BMA and the improvement of sustainable agriculture is mediated by soil health status and its enhancement. Moving from concept to practical application requires identifying which fields hold the greatest potential to advance sustainable agriculture, which offers more limited impact, and which specific combinations of approaches are most effective. By using a soil health index, multiple soil characteristics can be integrated and analyzed to estimate yield potential across different components of the system. In this context, we refer to soil health as an index that combines several biotic parameters, such as pathogen pressure and the abundance of symbiotic microbes, with abiotic parameters, including nutrient availability and soil organic carbon content^19,79^. Integrating the new BMA perspective with the well-established concept of soil health could facilitate broader and more rapid implementation.

**Fig. 3:**
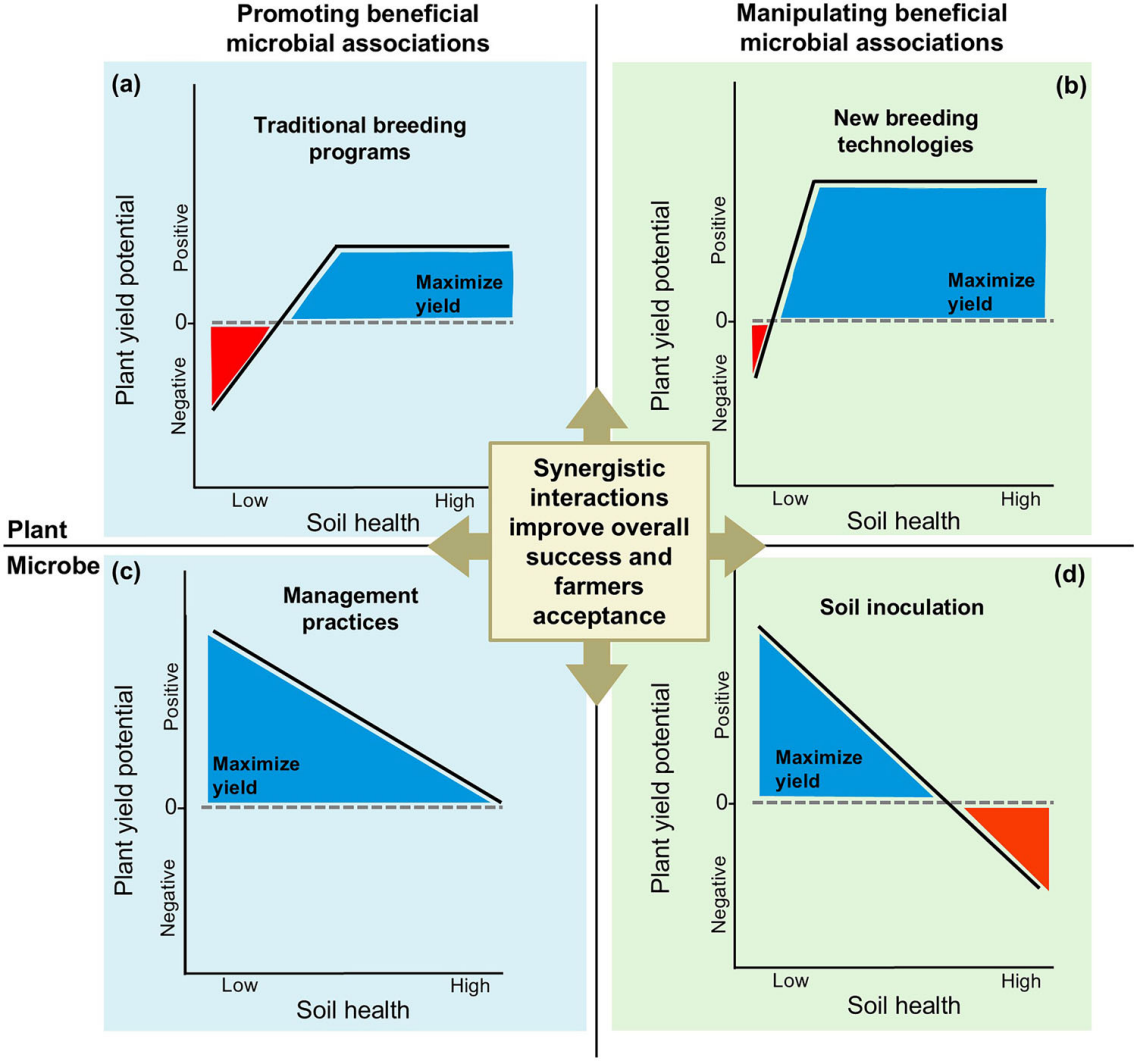
Schematic illustration of strategies to enhance beneficial microbial associations (BMA). The figure is organized along two axes: the horizontal axis separates plant-based interventions (top row) from microbial-based approaches (bottom row), while the vertical axis distinguishes strategies aimed at promoting beneficial associations (left) from those that manipulate associations (right). **a** **Top left – Traditional breeding:** Breeding programs that select crop genotypes with enhanced capacity to associate with beneficial microbes. In soils with very low health, beneficial microbes are scarce, and root traits that supposed to enhance BMA may have negative effects. Once soil health reaches a threshold, additional traits promoting beneficial associations show increasingly positive effects until they plateau. **b** **Bottom left – Management practices:** Agricultural practices that improve soil conditions and support native microbial communities. These follow a negative trend: in non-healthy soils, management practices can strongly boost beneficial interactions, whereas in already healthy soils, their effect is limited or neglectable. **c** **Top right – New Breeding Technologies (NBTs):** Approaches that enhance root traits or activate plant signaling pathways to attract specific microbial partners. These methods are especially effective for a large range of soil health gradients, where beneficial microbial populations are present. In poor soils, however, beneficial microbial abundance is too low, and excessive exudation may cause neutral or negative effects. Compared to traditional breeding, NBTs exhibit a steeper positive slope due to their targeted enhancement of plant–microbe signaling and smaller negative effects. **d** **Bottom right – Microbiome manipulation:** Direct inoculation of soils with beneficial microbes. This strategy is most effective in low-quality soils where native microbial communities are absent or depleted, but in already healthy soils, it can be redundant or even disruptive (Adapted from Rog et al., 2025, based on field AMF inoculation studies). The figure highlights the potential synergistic effect of using multiple approaches. For example- Soil inoculation and selection of plant- enhancing BMA specifically under low soil health.

From the microbial perspective, inoculation with BMA tends to have a stronger impact in less healthy soils compared to healthy ones (Fig. 3). This pattern has been observed in numerous field trials involving inoculation with AMF^79^. Similarly, certain management practices, such as reduced tillage, have shown a higher potential to improve plant yield in less healthy soils^55^ (Fig. 3).

On the plant side, however, available data is still limited, and research in this area is only beginning to develop^20^. Genotypes specifically bred for enhanced microbial associations will have an advantage in healthy soils, where beneficial microbes are already abundant. In contrast, in degraded or low-health soils, such genotypes may face a disadvantage, as traits important for microbial associations may not confer benefits and could even impose costs (Fig. 3). Plant breeding efforts might reach a plateau, where the contribution of naturally occurring beneficial microbes may already be saturated^80^. The application of NBTs could potentially strengthen BMA interactions and mitigate adverse effects under low soil health conditions by enhancing beneficial traits to a greater magnitude. Employing multiple, specifically designed NBT lines may achieve even higher levels of beneficial interaction. Integrating dual plant and soil bioengineering within the framework of soil health offers a holistic approach to advancing sustainable agriculture.

The interaction between plant-based and soil-based approaches represents a key aspect of a holistic strategy for improving agricultural sustainability. Our dual bioengineering framework emphasizes the essential link between plant and soil methods that promote beneficial plant–microbe associations. For instance, under low soil health conditions, combining soil management practices with the breeding of plants that possess beneficial traits can produce complementary effects (Fig. 3). Furthermore, integrating agricultural management techniques with plants developed through NBTs may further expand the range of success and yield improvements. Ideally, the combination of targeted soil inoculation and specific plant traits can be particularly effective. Overall, precision agriculture approaches that enhance plant–microbe interactions through the coordinated use of plant and soil bioengineering hold substantial potential for advancing sustainable crop production.

## Examples of dual bioengineering BMA field application

### Arbuscular mycorrhizal fungi and strigolactone association

AMF can enhance plant nutrient uptake and nutrient use efficiency, and also provide resistance to drought and diseases^12^. AMF are present in most healthy soils and can increase primary productivity in grasslands, and extend the water uptake zone of the root system and plant water relations^12,19^. However, in intensively fertilized and managed agricultural soils, AMF often have reduced abundance and diversity^81,82^. The combination of enrichment of the rhizosphere with AMF and using the plant genotypes with high complementarity to AMF has the potential to create beneficial and stable symbiosis and can promote natural processes, enabling agriculture to become more sustainable.

Exudation of the signaling molecule strigolactone (SL) promotes the interaction with AMF^83^. More than 40 different SLs have been identified across the plant kingdom, and individual plant species exude different blends of SLs from their roots into the rhizosphere, depending on plant species and variety^46^. In maize, SL biosynthesis could proceed through two pathways, mutations in genes from the pathway has been shown to change the flux through the pathways and alter the balance between the SL compounds^44^ (Supplementary Table 1). Interestingly, changes in the ratio between these different SLs affected the germination and attachment of the parasitic weed *Striga* to maize roots^44^. Other experiments have shown a high association of other SL molecules with root branching and mycorrhizal associations^84^. Thus, changes in the SL blend have a high potential to change the level of attractiveness of roots to beneficial AMF.

Root morphological and anatomical traits also play a critical role in facilitating and maintaining AMF interactions and can be used in breeding programs. Traits such as root order and, in particular, the presence of root hairs strongly influence microbial community composition and the establishment of symbiotic relationships^85^. Plant species with thick, sparsely branched root systems tend to exhibit higher colonization rates and greater abundance of AMF^86^. Moreover, the fungal community, especially AMF, often shows a preference for specific root types, such as axial versus lateral roots, which in turn affects the expression of phosphorus transporter genes^87^. Since *Striga* and AMF are both affected by SL levels, plants susceptible to *Striga* could be included in breeding programs to test for BMA. Integrating insights from both molecular signaling and root morphology presents a promising avenue for breeding plants with improved potential for beneficial microbial associations.

### Nitrogen fixing symbiotic bacteria and flavonoids

The efficient symbiotic association between plants and *Rhizobia* plays a vital role in the global nitrogen cycle and is especially important in intensively managed agricultural systems. Many farmers include nitrogen-fixing host plants in crop rotations to enrich soil nitrogen levels and improve overall land productivity^88^. This complex mutualistic interaction begins with the secretion of signaling molecules, flavonoids, by plant roots to attract *Rhizobia*, followed by a cascade of gene regulation that leads to symbiosis establishment and nodule formation^89^. Recent studies have identified key genes involved in the initiation of this endosymbiosis (Supplementary Table 1). The transfer of the pathway of such signaling molecules into crop species opens exciting opportunities to manipulate and enhance plant-*Rhizobia* associations through genetic and biotechnological approaches.

To date, more than 8000 flavonoid compounds have been identified across various plant species^90^. These compounds are synthesized in multiple plant organs, and their core structures can be extensively modified to produce a wide array of products with diverse biological functions, mobility profiles, and signaling capacities. While the biosynthesis of flavonoids is well understood in many crops, the exudation of the molecules to the rhizosphere is less well understood, although some progress has been made recently in identifying transporters^91^. The flavonoids could play a role in the selection of compatible rhizobia species by the host^92^ and lead to modification of the associated microbial community. The host plant attempts to select the most beneficial symbiotic nitrogen-fixing bacteria^93^. At the same time, some studies found beneficial effects with multi-strain associations^94^, while others present more efficient symbiosis interaction with individual strains^95^.

Flavonoids are not restricted to attracting N-fixing bacteria but also to attracting AMF, PGPB, and acting as defense molecules against root pathogens. Since nodule formation is a relatively simple phenotypic trait for breeders to evaluate, it could be used for large-scale selection, while flavonoid profiling could be reserved for a smaller subset of selected genotypes. Given that flavonoids also participate in complex signaling interactions within the rhizosphere, their broader ecological roles warrant further research.

### Attraction of plant-promoting bacteria by benzoxazinoids

Benzoxazinoids are secondary metabolites that regulate beneficial associations, specifically PGPB. These signaling compounds are exuded by grasses and cereal crops commonly used in agriculture. In addition to functioning as a chemoattractant, benzoxazinoids contribute to pathogen defense and modulate the structure and function of the rhizosphere microbiome^96,97^. Remarkably, benzoxazinoid exudation in cereals has been shown to shift microbial community composition toward a higher abundance of BMA, even influencing the microbiome of subsequent crops in a rotation system. This legacy effect not only enhances plant performance but can also influence plant–herbivore interactions^98^. Breeding programs should consider crop rotation cycles, including the sequence of different crops, during both candidate selection and the design of breeding strategies. Other morphological traits can enhance association with PGPB, such as increased numbers and lengths of lateral roots. Manipulating the levels of Benzoxazinoids using NBT presents a promising strategy to promote BMA in both current and future cropping cycles.

### Suppression of negative biological activities

While some exudates attract beneficial microbes, others suppress biological activities that influence nutrient accessibility. Biological nitrification inhibition (BNI) is an important example of how root exudates can regulate soil processes to enhance nutrient availability^99^. In agricultural systems, ammonium is a major nitrogen source applied by farmers, but it is rapidly converted into nitrate, a highly mobile form that is more susceptible to leaching. BNI compounds released by plant roots inhibit nitrifying microorganisms, slowing this conversion and helping retain plant-available nitrogen. The presence of BNI activity in certain plant species, with particularly high capacity identified in wild relatives of wheat, making it a promising target for crop improvement. Importantly, BNI has been shown to function not only against isolated bacterial strains but also within complex soil microbial communities, increasing its relevance for real-world agricultural systems^100^. More recently, the transfer of chromosomal regions associated with BNI from wheat relatives into cultivated wheat has led to improved biomass production and grain yield^99^. When applying BNI, it is important to verify the absence of any non-target effects on other beneficial microbes. Integrating crop genotypes with enhanced BNI capacity, potentially through NBT, represents a promising strategy to improve nitrogen use efficiency and support more sustainable, low-input agricultural systems.

## Framework for future research and practical directions

### Predication models for beneficial microbial associations

Currently, there is no widely accepted predictive model to estimate the effectiveness of BMA inoculation, which remains highly variable across different crop types, soil conditions, and field environments. Developing such models is crucial for predicting outcomes in specific crop–microbe combinations and optimizing inoculation strategies to maximize yield and microbial synergy (Fig. 1). Recently, a significant advancement was made with the development of the first field-based predictive model for AMF inoculation. This model demonstrated that maize yield response to AMF inoculation could be best predicted by the presence of soil-borne fungal pathogens^31,79^. The model successfully predicted over 80% of the variation in plant growth in response to inoculation. Experimental studies need to confirm such findings. Another recent prediction model, based on 240 fields, provided variety-specific relationships between the seed potato tuber microbiome and next season’s crop vigor^101^. Future BMA prediction models should incorporate soil microbiome composition, plant genotype, soil and environmental variables, and microbial traits to support precision agriculture and tailored plant-BMA practices.

Prediction models for BMA must be crop-specific, much like existing models for irrigation, fertilization, and harvesting. Each crop exhibits unique biological characteristics, growth patterns, and pathogen susceptibilities that influence the success of microbial inoculation (Fig. 2). Therefore, testing and validating BMA models must include crop-relevant parameters, such as prevalent fungal pathogens (e.g., *Fusarium tritici* in wheat) and species-specific developmental traits. To address this complexity, artificial intelligence (AI) and machine learning approaches offer powerful tools for analyzing large, multidimensional datasets. These models can predict microbial inoculation outcomes across both short- and long-term scales and under a variety of environmental conditions^102^. Such an application should allow users to input field-specific data, such as soil nutrient profiles, microbial community composition, pathogen loads, and environmental conditions, and return a prediction of BMA effectiveness^103^. Next-generation prediction tools can help farmers anticipate outcomes, optimize BMA use, and ensure more consistent soil and crop health across diverse agroecosystems.

### Using new plant-microbe signaling to enhance BMA association

Plants likely influence the microbial rhizosphere through a whole array of small molecules, many of which remain to be discovered^46^. For example, small plant peptides (SPPs) have been shown to shape the rhizosphere microbiome by modifying microbial gene expression, root morphology, and plant immune responses^104^. Future breeding efforts may focus on enhancing bidirectional signaling pathways that attract specific microbial partners to both the rhizosphere and the phyllosphere^105^. In addition, plants secrete extracellular vesicles (EVs) to transport biomolecules across long distances^106^. Certain EVs have been shown to mediate plant–microbe communication and even contribute to the formation of nitrogen-fixing nodules. While many biomolecules are believed to be transferred via EVs, the mechanisms underlying their formation, cargo selection, and roles in plant-microbe interactions are still being uncovered, offering new opportunities for breeding innovations. Another promising signaling mechanism involves small RNA, which can be transferred via vesicles or directly into the rhizosphere. Cross-kingdom RNAi has recently been shown to regulate endosymbiotic associations and trigger immune responses^107^. While these signaling pathways are still under active search, they represent exciting frontiers for breeding strategies aimed at enhancing beneficial microbial interactions.

Future directions for breeding traits that promote BMA include enhancing carbon allocation to belowground compartments, particularly in ways that support microbial interactions. Plants can adjust carbon distribution in response to environmental stress, but the specific signaling cascades and regulatory mechanisms involved remain major open questions in plant science. Recent studies have shown that under drought conditions, plants allocate more carbon belowground, especially through increased root exudation^108^. Understanding the transport systems for soluble sugars, as well as the regulation of starch metabolism, could offer promising gene targets for BMA-focused breeding programs. For example, SWEET transporters, which mediate the movement of mono- and disaccharides, represent key candidates for carbohydrate manipulation^51^. In parallel, new breeding efforts are exploring plants with deeper root systems and specific microbial associations in deeper soil layers^109^. However, further research is needed to precisely identify the molecular mechanisms that control belowground carbon allocation—at the whole-plant, cellular, and rhizosphere levels.

### The phyllosphere as another compartment for breeding for BMA

The phyllosphere microbiome is an emerging field with significant potential for targeted manipulation in agriculture. The phyllosphere microbiome can enhance plant performance under a range of biotic and abiotic stress conditions^110^. Several studies have demonstrated direct antagonistic interactions between phyllosphere microbes and plant pathogens, contributing to host defense^111^. As in the rhizosphere, regulatory mechanisms underlying microbiome homeostasis in the phyllosphere involve secondary metabolites^34^. However, in the phyllosphere, available evidence suggests that *M* genes primarily promote the enrichment of disease-suppressive microbiota and associated disease suppression, rather than directly enhancing BMA, as has been reported for the rhizosphere. Developing such cooperative microbiome assemblies could provide a more holistic strategy for enhancing BMA both in the rhizosphere and the phyllosphere and advancing sustainable agricultural systems.

### The potential of socio-political and socio-economic acceptance

An important condition for the successful diffusion of novel agri-food technologies is their socio-political and socio-economic acceptance, with the politicization of these technologies being a potential barrier to scaling up innovations^112–114^. With respect to new breeding and soil inoculation technologies, more systematic empirical evidence on the acceptance among key stakeholder groups, such as farmers and citizens, is needed. Existing research has focused more on citizens’ than farmers’ attitudes toward NBT plants and microbial based agriculture solutions. For instance, Swiss citizens are skeptical about the use of NBT plants and prefer strict regulations^115,116^. However, a recent study^117^ indicates that consumers from the United States are more open to NBT in plant breeding and agriculture, like CRISPR-Cas9, than Swiss consumers. This study also highlights that, besides the cultural also the regulatory context might function as a risk cue to citizens when forming an opinion on NBT (Fig. 2). Previous findings from the US indicate that genetically modified organism (GMO) adoption is primarily driven by perceived economic benefits, such as increased yields and reduced input costs, particularly in pest and weed management^118,119^. with strict regulatory frameworks or strong consumer opposition, as seen in parts of Europe^119^. While there is some evidence of farmers’ acceptance of GMO plants in general, more research on the acceptance of novel NBT plants is necessary to close important research gaps.

When microbial technologies are combined with NBT and non-NBT plants, the acceptance dynamics become even more complex, and less evidence exists. While stakeholders like farmers potentially value microbial technologies for their potential environmental benefits and potential to reduce chemical and fertilizer inputs, their effectiveness can be variable, and their integration into existing farming systems presents practical challenges that require targeted behavioral interventions and information to increase adoption rates^120–122^ (Fig. 2). Moreover, combining microbial technologies with NBT introduces additional considerations, such as regulatory compliance, consumer perceptions, and ethical concerns related to the use of biotechnology in agriculture. Farmers’ willingness to adopt these combined technologies is, therefore, likely to be influenced by a multitude of factors, including the perceived reliability and effectiveness of microbial products, beliefs and social norms, the perceived economic and environmental benefits, and the compatibility of these technologies with their current farming practices^123^. Understanding these complex factors and their relative importance is crucial for developing strategies promoting sustainable adoption of innovative agricultural technologies.

In sum, studying both citizens and farmer attitudes – as well as attitudes of further key stakeholders in agri-food supply chains, such as processors, retailers, and regulators – is a key prerequisite to not only assess the potential effectiveness of different complementary plant- and microbe-focused strategies to improve soil health but also their socio-economic and socio-political feasibility.

## Supplementary information

Supplementary Information
