# Supplementary material for: Breeding for beneficial microbial associations
Source: Nat Commun. 2026 Aug 3;17:7695. doi: 10.1038/s41467-026-76260-6 (PMC13434687; doi:10.1038/s41467-026-76260-6)
Supplement: Supplementary file 1 [file 41467_2026_76260_MOESM1_ESM.pdf]

Supplementary Information

Breeding for beneficial microbial associations

Ido Rog[1,2], Stefanie Lutz[1,2] Lukas Fesenfeld[3], Jürg Hiltbrunner[4], Harro Bouwmeester[5], Marcel G. A. van der Heijden[1,2]

Supplementary tables- Supplementary Table 1 and Supplementary Table 2.

Supplementary Table 1. List of plant *Microbiome genes* (*M* genes) or loci for targeted breeding to potentially enhance beneficial associations. The gene targets can be used for classical breeding selection as QTLs or for generating plants with DNA editing using NBT methods. Names of genes/loci can be changed according to the organism; we present the crop species, the function, and the relevant microbe. Examples containing the potential to enhance *M* genes candidate, but further experiments are needed to show the function.

Gene / Locus	Crop Species	Function	Associated Microbes	Reference
CAROTENOID CLEAVAGE DIOXYGENASE 7 and 8a	*Zea mays, Solanum lycopersicum and others*	Strigolactone biosynthesis; promotes AMF colonization	AMF	[1–3]
**PDR1**	*Petunia hybrida, Solanum lycopersicum*	SL transporter for root exudation	AMF	[4,5]
**D14L (DWARF14-LIKE)**	*Oryza sativa*	Perceives fungal signals; required for AMF colonization	AMF	[6]
**CCaMK (OsCCaMK)**	*Oryza sativa*	Symbiotic calcium signaling protein; required for AMF colonization	AMF	[7,8]
**SYMRK (OsSYMRK)**	*Oryza sativa*	Symbiosis receptor kinase; conserved signaling component	AMF	[9]
**MYB72**	*Triticum aestivum*, *Arabidopsis thaliana*	Regulates root exudates & PGPR recruitment	PGPR	[10]
**NSP1 / NSP2**	*Glycine max*, legumes	Transcription factors required for nodulation & AMF gene expression, incl SL biosynthesis	AMF, Rhizobia	[11,12]
**NIN**	*Glycine max*, *Medicago*	Nodule initiation factor; key for rhizobia infection	Rhizobia	[13]
**NFR1 NFR5**	*Glycine max*, *Medicago*	LysM receptors for Nod factor perception	Rhizobia	[14]
**DELLA proteins**	*Triticum aestivum*, *Oryza sativa*	Repress GA signaling, positively regulate AMF colonization	AMF	[15]
**OsCERK1**	*Oryza sativa*	Receptor kinase, balancing defense and symbiosis	AMF, PGPR	[16]
**EXO70I**	*Medicago truncatula*	Exocyst complex; required for arbuscule development	AMF	[17]
**CNGC15**	*Medicago truncatula*	symbiotic-induced nuclear Ca^{2+} oscillations, flavenoide cholesterol	Rhizobia, AMF	[18]
**flavonoid 3'-hydroxylases**	*Oryza sativa*	Flavone biosynthetic pathway to induce biofilm formation in soil diazotrophs	Diazotrophs, free living N fixing bacteria	[19]

**PHOSPHATE STARVATION RESPONSE 2 (PHR2)**	*Oryza sativa*	Required for pre-contact signaling, colonization, and function	AMF	20
**SlSPX1 (SYG1 (suppressor of yeast GPA1)/Pho81(phosphate 81)/XPR1 (xenotropic and polytropic retrovirus receptor 1)**	*Solanum lycopersicum*	SlSPX1-SlPHRs form a Pi-sensing module to coordinate the AM symbiosis	AMF	21
ZmNPF4.5	*Zea mays*	Mycorrhizal NO3− acquisition, a symbiotic N uptake route	AMF	22,23
PHT1	*AMF host*	Regulation of phosphorus homeostasis localize on the plasma membrane	AMF	24
WRINKLED 5a (WRI5a)	*Medicago truncatula*	Transcription Factor, a Master Regulator of Lipid Biosynthesis and Transfer during Mycorrhizal Symbiosis	AMF	25
LONGATED HYPOCOTYL5 (HY5)	*Solanum lycopersicum*	Light-dependent activation of HY5 promotes mycorrhizal symbiosis by SL biosynthesis	AMF	2
carotenoid cleavage dioxygenase OsZAS	*Oryza sativa*	Produce zaxinone, regulatory network that involves SL	AMF	26
rhizopine scyllo-inosamine	*Medicago truncatula and Hordeum vulgare*	Synthetic signaling networks between plants and bacteria	bacteria	27
DMI1 (Does not Make Infections 1) and its homologs CASTOR and POLLUX	*Lotus japonicus*	Highly selective Ca^{2+} channel whose activation requires cytosolic/nucleosolic Ca^{2+}	AMF/ Rhizobia	28
REQUIRED FOR ARBUSCULAR MYCORRHIZATION1 (RAM1)	*Solanum lycopersicum*	Transcription factor from the GRAS family, master regulator	AMF	29
BENZOXAZINELESS1 (BX1) or INDOLE GLYCEROL PHOSPHATE LYASE (IGL)	*Zea mays*	Benzoxazinoids biosynthetic levels led to accumulation of DIMBOA functions as a defense regulatory signal	Bacteria	30
FIXATION UNDER NITRATE (FUN)	*Lotus japonicus*	Deactivation might increase N-fixation	Rhizobia	31

Supplementary Table 2. Examples of agriculture practices and their effect on beneficial microbial association (BMA).

Practice	Effect on Beneficial Microbes	Details	References
**Tillage**	Negative	Disrupts soil structure, fungal hyphae, especially damaging to AMF networks.	32–34
**Crop Rotation**	Positive	Enhance microbial diversity, breaks pathogen cycles, supports diverse PGPR and AMF communities.	35–37
**Cover Cropping**	Positive	Increases organic matter, root exudates feed microbes, promotes AMF and nitrogen-fixing bacteria.	38–40
**Synthetic Fertilizers (N, P, K)**	Mixed / Often negative	High phosphorus and nitrogen reduce AMF and Rhizobia colonization respectively	41,42
**Compost application**	Positive	Enhances microbial activity and diversity; improves habitat for PGPR and saprophytic fungi.	43,44
**Pesticide Use**	Negative / Mixed	Many pesticides are non-selective and reduce microbial biomass and diversity, including beneficials. Under certain conditions, pesticides might be metabolized by microbes and increase microbial biomass.	45–47
**Irrigation Practices**	Variable	Moderate irrigation supports microbes. Improve microbe distribution	48–50
**Intercropping of different varieties**	Positive	Complementarity effects and yield benefits intercropping maize or wheat varieties. Different root structures, pathogen resistance levels and maturation.	51,52
**Intercropping of different plant species**	Mixed / Often positive	Mixing plants from different functional groups such as legumes improves N content, and soil niche partitioning by combining deep/shallow plants increases yield but reduces harvest index.	53–56

Supplementary References

1. Akiyama, K., Matsuzaki, K. & Hayashi, H. Plant sesquiterpenes induce hyphal branching in arbuscular mycorrhizal fungi. *Nature* **435**, 824–827 (2005).
2. Ge, S. *et al.* Light-dependent activation of HY5 promotes mycorrhizal symbiosis in tomato by systemically regulating strigolactone biosynthesis. *New Phytologist* **233**, 1900–1914 (2022).
3. Bouwmeester, H., Dong, L., Wippel, K., Hofland, T. & Smilde, A. The chemical interaction between plants and the rhizosphere microbiome. *Trends Plant Sci.* (2025).
4. Kretzschmar, T. *et al.* A petunia ABC protein controls strigolactone-dependent symbiotic signalling and branching. *Nature* **483**, 341–344 (2012).
5. Ban, X. *et al.* Manipulation of a strigolactone transporter in tomato confers resistance to the parasitic weed broomrape. *The Innovation* **6**, 100815 (2025).
6. Gutjahr, C. *et al.* Rice perception of symbiotic arbuscular mycorrhizal fungi requires the karrikin receptor complex. *Science (1979).* **350**, 1521–1524 (2015).
7. Hayashi, T. *et al.* A dominant function of CCaMK in intracellular accommodation of bacterial and fungal endosymbionts. *The Plant Journal* no-no (2010) doi:10.1111/j.1365-313X.2010.04228.x.
8. Singh, S., Katzer, K., Lambert, J., Cerri, M. & Parniske, M. CYCLOPS, A DNA-Binding Transcriptional Activator, Orchestrates Symbiotic Root Nodule Development. *Cell Host Microbe* **15**, 139–152 (2014).
9. Yoshida, S. & Parniske, M. Regulation of Plant Symbiosis Receptor Kinase through Serine and Threonine Phosphorylation. *Journal of Biological Chemistry* **280**, 9203–9209 (2005).
10. Stringlis, I. A. *et al.* Root transcriptional dynamics induced by beneficial rhizobacteria and microbial immune elicitors reveal signatures of adaptation to mutualists. *The Plant Journal* **93**, 166–180 (2018).
11. Gobbato, E. *et al.* A GRAS-Type Transcription Factor with a Specific Function in Mycorrhizal Signaling. *Current Biology* **22**, 2236–2241 (2012).
12. Hirsch, S. *et al.* GRAS Proteins Form a DNA Binding Complex to Induce Gene Expression during Nodulation Signaling in *Medicago truncatula* . *Plant Cell* **21**, 545–557 (2009).
13. Liu, C.-W. *et al.* A protein complex required for polar growth of rhizobial infection threads. *Nat. Commun.* **10**, 2848 (2019).
14. Radutoiu, S. *et al.* Plant recognition of symbiotic bacteria requires two LysM receptor-like kinases. *Nature* **425**, 585–592 (2003).
15. Floss, D. S., Levy, J. G., Lévesque-Tremblay, V., Pumplin, N. & Harrison, M. J. DELLA proteins regulate arbuscule formation in arbuscular mycorrhizal symbiosis. *Proceedings of the National Academy of Sciences* **110**, (2013).

16. Miyata, K. *et al.* The Bifunctional Plant Receptor, OsCERK1, Regulates Both Chitin-Triggered Immunity and Arbuscular Mycorrhizal Symbiosis in Rice. *Plant Cell Physiol.* **55**, 1864–1872 (2014).

17. Zhang, X., Pumplin, N., Ivanov, S. & Harrison, M. J. EXO70I Is Required for Development of a Sub-domain of the Periarbuscular Membrane during Arbuscular Mycorrhizal Symbiosis. *Current Biology* **25**, 2189–2195 (2015).

18. Cook, N. M. *et al.* Autoactive CNGC15 enhances root endosymbiosis in legume and wheat. *Nature* **638**, 752–759 (2025).

19. Yan, D. *et al.* Genetic modification of flavone biosynthesis in rice enhances biofilm formation of soil diazotrophic bacteria and biological nitrogen fixation. *Plant Biotechnol. J.* **20**, 2135–2148 (2022).

20. Das, P. P. *et al.* Plant-soil-microbes: A tripartite interaction for nutrient acquisition and better plant growth for sustainable agricultural practices. *Environ. Res.* **214**, 113821 (2022).

21. Liao, D. *et al.* SlSPX1-SlPHR complexes mediate the suppression of arbuscular mycorrhizal symbiosis by phosphate repletion in tomato. *Plant Cell* **34**, 4045–4065 (2022).

22. Wang, S. *et al.* Functional analysis of the OsNPF4.5 nitrate transporter reveals a conserved mycorrhizal pathway of nitrogen acquisition in plants. *Proceedings of the National Academy of Sciences* **117**, 16649–16659 (2020).

23. Wang, S. *et al.* OsNLP3 and OsPHR2 orchestrate direct and mycorrhizal pathways for nitrate uptake by regulating NAR2.1–NRT2s complexes in rice. *Proceedings of the National Academy of Sciences* **122**, (2025).

24. Zhao, B. *et al.* Microbe-dependent and independent nitrogen and phosphate acquisition and regulation in plants. *New Phytologist* **242**, 1507–1522 (2024).

25. Jiang, Y. *et al.* Medicago AP2-Domain Transcription Factor WRI5a Is a Master Regulator of Lipid Biosynthesis and Transfer during Mycorrhizal Symbiosis. *Mol. Plant* **11**, 1344–1359 (2018).

26. Votta, C. *et al.* Zaxinone synthase controls arbuscular mycorrhizal colonization level in rice. *The Plant Journal* **111**, 1688–1700 (2022).

27. Geddes, B. A. *et al.* Engineering transkingdom signalling in plants to control gene expression in rhizosphere bacteria. *Nat. Commun.* **10**, 3430 (2019).

28. Kim, S. *et al.* Ca2+-regulated Ca2+ channels with an RCK gating ring control plant symbiotic associations. *Nat. Commun.* **10**, 3703 (2019).

29. Ho-Plágaro, T., Tamayo-Navarrete, M. I., Ćavar Zeljković, S., Tarkowski, P. & García-Garrido, J. M. A dual regulatory role for the arbuscular mycorrhizal master regulator RAM1 in tomato. *J. Exp. Bot.* **75**, 5021–5036 (2024).

30. Ahmad, S. *et al.* Benzoxazinoid Metabolites Regulate Innate Immunity against Aphids and Fungi in Maize . *Plant Physiol.* **157**, 317–327 (2011).

31. Lin, J. *et al.* Zinc mediates control of nitrogen fixation via transcription factor filamentation. *Nature* **631**, 164–169 (2024).

32. Kabir, Z. Tillage or no-tillage: Impact on mycorrhizae. *Canadian Journal of Plant Science* **85**, 23–29 (2005).

33. Jansa, J. and M. A. and K. G. and A. T. and R. R. and S. I. R. and F. E. SOIL TILLAGE AFFECTS THE COMMUNITY STRUCTURE OF MYCORRHIZAL FUNGI IN MAIZE ROOTS. *Ecological Applications,* (2003).

34. Wittwer, R. A., Dorn, B., Jossi, W. & van der Heijden, M. G. A. Cover crops support ecological intensification of arable cropping systems. *Sci. Rep.* **7**, 41911 (2017).

35. Schlatter, D. C., Bakker, M. G., Bradeen, J. M. & Kinkel, L. L. Plant community richness and microbial interactions structure bacterial communities in soil. *Ecology* **96**, 134–142 (2015).

36. Hartman, K. *et al.* Cropping practices manipulate abundance patterns of root and soil microbiome members paving the way to smart farming. *Microbiome* **6**, 14 (2018).

37. Vukicevich, E., Lowery, T., Bowen, P., Úrbez-Torres, J. R. & Hart, M. Cover crops to increase soil microbial diversity and mitigate decline in perennial agriculture. A review. *Agron. Sustain. Dev.* **36**, 48 (2016).

38. Tiemann, L. K., Grandy, A. S., Atkinson, E. E., Marin-Spiotta, E. & McDaniel, M. D. Crop rotational diversity enhances belowground communities and functions in an agroecosystem. *Ecol. Lett.* **18**, 761–771 (2015).

39. De Deyn, G. B., Cornelissen, J. H. C. & Bardgett, R. D. Plant functional traits and soil carbon sequestration in contrasting biomes. *Ecol. Lett.* **11**, 516–531 (2008).

40. Wittwer, R. A. & van der Heijden, M. G. A. Cover crops as a tool to reduce reliance on intensive tillage and nitrogen fertilization in conventional arable cropping systems. *Field Crops Res.* **249**, 107736 (2020).

41. Johnson, N. C. Can Fertilization of Soil Select Less Mutualistic Mycorrhizae? *Ecological Applications* **3**, 749–757 (1993).

42. Treseder, K. K. A meta-analysis of mycorrhizal responses to nitrogen, phosphorus, and atmospheric CO_2 in field studies. *New Phytologist* **164**, 347–355 (2004).

43. Lazcano, C., Gómez-Brandón, M., Revilla, P. & Domínguez, J. Short-term effects of organic and inorganic fertilizers on soil microbial community structure and function. *Biol. Fertil. Soils* **49**, 723–733 (2013).

44. Bonanomi, G., Antignani, V., Capodilupo, M. & Scala, F. Identifying the characteristics of organic soil amendments that suppress soilborne plant diseases. *Soil Biol. Biochem.* **42**, 136–144 (2010).

45. Cycoń, M. & Piotrowska-Seget, Z. Pyrethroid-Degrading Microorganisms and Their Potential for the Bioremediation of Contaminated Soils: A Review. *Front. Microbiol.* **7**, (2016).

46. Imfeld, G. & Vuilleumier, S. Measuring the effects of pesticides on bacterial communities in soil: A critical review. *Eur. J. Soil Biol.* **49**, 22–30 (2012).

47. Walder, F. *et al.* Soil microbiome signatures are associated with pesticide residues in arable landscapes. *Soil Biol. Biochem.* **174**, 108830 (2022).

48. Chen, F., Ronen, Z. & Arye, G. Spatial distribution of *Azospirillum brasilense* in <scp>2D</scp> flow cell: Effect of irrigation and inoculation regimes. *Eur. J. Soil Sci.* **75**, (2024).

49. Calvo, A. *et al.* Interactions between an arbuscular mycorrhizal inoculum and the root-associated microbiome in shaping the response of Capsicum annuum “Locale di Senise” to different irrigation levels. *Plant Soil* **508**, 361–383 (2025).

50. Liu, R. J., Sheng, P. P., Hui, H. Bin, Lin, Q. & Chen, Y. L. Integrating irrigation management for improved grain yield of winter wheat and rhizosphere AM fungal diversity in a semi-arid cropping system. *Agric. Syst.* **132**, 167–173 (2015).

51. Wang, X., Hoffland, E., Feng, G. & Kuyper, T. W. Arbuscular mycorrhizal symbiosis increases phosphorus uptake and productivity of mixtures of maize varieties compared to monocultures. *Journal of Applied Ecology* **57**, 2203–2211 (2020).

52. Stefan, L. *et al.* Multi-trait assessment of wheat variety mixtures performance and stability: Mixtures for the win! *European Journal of Agronomy* **164**, 127504 (2025).

53. Hage-Ahmed, K., Krammer, J. & Steinkellner, S. The intercropping partner affects arbuscular mycorrhizal fungi and Fusarium oxysporum f. sp. lycopersici interactions in tomato. *Mycorrhiza* **23**, 543–550 (2013).

54. Chamkhi, I. *et al.* Legume-based intercropping systems promote beneficial rhizobacterial community and crop yield under stressing conditions. *Ind. Crops Prod.* **183**, 114958 (2022).

55. Bargaz, A. *et al.* Species interactions enhance root allocation, microbial diversity and P acquisition in intercropped wheat and soybean under P deficiency. *Applied Soil Ecology* **120**, 179–188 (2017).

56. Stefan, L., Hartmann, M., Engbersen, N., Six, J. & Schöb, C. Positive Effects of Crop Diversity on Productivity Driven by Changes in Soil Microbial Composition. *Front. Microbiol.* **12**, (2021).
